# Acute melatonin administration improves exercise tolerance and the metabolic recovery after exhaustive effort

**DOI:** 10.1038/s41598-021-97364-7

**Published:** 2021-09-28

**Authors:** Vinícius Silva Faria, Taciane Maria Melges Pejon, Claudio Alexandre Gobatto, Gustavo Gomes de Araujo, Anabelle Silva Cornachione, Wladimir Rafael Beck

**Affiliations:** 1 Laboratory of Endocrine Physiology and Physical Exercise, Department of Physiological Sciences, Federal University of São Carlos, São Carlos, SP 13565-905 Brazil; 2 Laboratory of Applied Sport Physiology, School of Applied Sciences, University of Campinas, Limeira, SP 13484-350 Brazil; 3 Muscle Physiology and Biophysics Laboratory, Department of Physiological Sciences, Federal University of São Carlos, São Carlos, SP 13565-905 Brazil

**Keywords:** Physiology, Endocrinology

## Abstract

The present study investigated the effects of acute melatonin administration on the biomarkers of energy substrates, GLUT4, and FAT/CD36 of skeletal muscle and its performance in rats subjected to exhaustive swimming exercise at an intensity corresponding to the maximal aerobic capacity (*t*lim). The incremental test was performed to individually determine the exercise intensity prescription and 48 h after, the animals received melatonin (10 mg·kg^−1^) or vehicles 30 min prior to *t*lim. Afterwards, the animals were euthanized 1 or 3 h after the exhaustion for blood and muscles storage. The experiment 1 found that melatonin increased the content of glycogen and GLUT4 in skeletal muscles of the animals that were euthanized 1 (p < 0.05; 22.33% and 41.87%) and 3 h (p < 0.05; 37.62% and 57.87%) after the last procedures. In experiment 2, melatonin enhanced the *t*lim (p = 0.01; 49.42%), the glycogen content (p < 0.05; 40.03%), GLUT4 and FAT/CD36 in exercised skeletal muscles (F = 26.83 and F = 25.28, p < 0.01). In summary, melatonin increased energy substrate availability prior to exercise, improved the exercise tolerance, and accelerated the recovery of muscle energy substrates after the *t*lim, possibly through GLUT4 and FAT/CD36.

## Introduction

The regulatory role of exogenous melatonin (N-acetyl-5-methoxytryptamine; molecular weight: 232 kDa) in circadian and seasonal rhythms has been well established^1^; nevertheless, there is growing evidence that broadly demonstrates several other functions, including antioxidant properties^2–5^, anti-inflammatory effects^6^, changes in energy metabolism^7^, the prevention and/or inhibition of cancer development^8,9^, and the treatment of neurological diseases^10^, diabetes^7,11^, sleep disorders^12^, and obesity^7,13^. Beyond these features, studies have also demonstrated the ergogenic effects of melatonin on performance in physical exercise^14,15^.

Physical exercise depends on the intermediary metabolism for ATP resynthesis—mainly through the chemical transformation of carbohydrates and lipids—as the intramuscular ATP concentration limits (~ 5 mmol per kg of wet muscle) the contractile activity for extended periods^16^. A substantial increase in muscle glucose uptake is fundamental in sustaining the energy needed for endurance exercise^17^, and this occurs through facilitated diffusion, which is carried out by the translocation of the glucose transporter (GLUT4) to the sarcolemma and transverse tubes^18^. A single physical exercise session can increase the GLUT4 content^11,19–21^, thus improving glucose uptake and the consequent oxidation or glycogen storage during recovery. As the duration of the exercise increases, a greater supply of substrates is necessary, such as carbohydrates from the liver or intestine, free fatty acids (FFAs) released from adipose tissue, and intramuscular triglycerides^22–24^. Thus, endurance exercise requires an enhanced pool of fatty acid translocase CD36 (FAT/CD36) in the sarcolemma and mitochondrial membrane in order to increase the uptake and oxidation rate of FFAs^25–27^. A single physical exercise session is also known to increase FAT/CD36 in the skeletal muscle of rats^28^, thus possibly influencing the energy metabolism for the next exercise session. In this scenario, some studies have demonstrated the effects of melatonin by increasing the content of glycogen in the muscles and liver and altering the bioavailability of blood glucose and plasma free fatty acids after long-term acute exercise^29–32^.

Compelling evidence has shown carbohydrate dependence during high-intensity, long-term exercise^16,22,33^ and that its reduction is a limiting factor for performance^34^. Therefore, considering the modulating role of melatonin in energy metabolism^32^ and its ability to increase performance in long-term physical exercises^14,15^, it is necessary to study its influence on glucose and free fatty acid transporters in skeletal muscle—beyond the substrates themselves—in order to confirm and better understand the mechanism of the ergogenic effect. In addition, if melatonin positively influences the GLUT4 and FAT/CD36 in exercised muscle, it could accelerate the metabolic recovery, which would be a considerable advantage in future efforts, at least from the bioenergetic point of view. Nevertheless, no studies have shown the effects of melatonin administration on the content of energy substrates and their transporters (GLUT4 and FAT/CD36) in skeletal muscle several hours after a long-term exercise session. Thus, the present study aimed to investigate the effects of melatonin on the energy substrates in the plasma and muscle, as well as GLUT4 and FAT/CD36 in the skeletal muscle, in rats that were subjected to exhaustive swimming exercise at an intensity corresponding to the maximal aerobic capacity. We hypothesized that melatonin administration increases the GLUT4, FAT/CD36, and energy substrates in exercised skeletal muscle, thus enhancing performance in terms of endurance and metabolic recovery.

## Methods

### Animals and environmental conditions

Sixty-eight male *Rattus norvegicus albinus* (Wistar) rats that were 45 days old were housed in a bioterium and kept in polypropylene cages (length: 40 cm, width: 40 cm, height: 20 cm, and 5 animals per cage); they received feed and water ad libitum. Throughout the experiment, the environmental conditions were maintained, including the temperature (22 ± 2 ºC), relative humidity (45% and 55%), noise (< 85 decibels), and photoperiod (10:14 h light/dark cycle). Incandescent lamps (Philips’s brand, soft model, 100 W, 2700 K; 565–590 nm; 60 lx, measured with a lux meter) were used during the 10-h light cycle. To carry out experimental interventions with the rats during the dark cycle (nighttime: 4:00 pm to 6:00 am), reflectors were installed in the bioterium and room and were surrounded by a red filter (ROSCO brand, model # fire19; > 600 nm; < 15 lx)^14,15,35^. The experimental procedures were conducted in accordance with Ethical Principles in Animal Research, adopted by the Brazilian College of Animal Experimentation (COBEA, Brazil) and was approved by the Ethics Committee on the Use of Animals (CEUA) of Federal University of São Carlos (São Paulo, Brazil) under protocol no. 9144181218. The experimental procedures were conducted in accordance with the Ethical Principles in Animal Research (ARRIVE guidelines 2.0).

### Experimental design

The animals (n = 68) were randomly split into 7 groups: a control (Ct: n = 10), rats treated with melatonin and euthanized 1 h (M1: n = 9) or 3 h after the last procedures (M3: n = 9), rats that exercised and were euthanized 1 h (Ex1: n = 10) or 3 h after the time to exhaustion (*t*lim) (Ex3: n = 10), and rats that were treated with melatonin, exercised, and were euthanized 1 h (ME1: n = 10) or 3 h after *t*lim (ME3: n = 10). The animals in the Ct, Ex1, and Ex3 groups received vehicles, while the animals in the M1, ME1, M3, and ME3 groups received melatonin (10 mg·kg^−1^); both were administered in the same volume. The M1, Ex1, and ME1 groups and the M3, Ex3, and ME3 groups were euthanized 1 and 3 h after the end of the experimental procedures for each group, respectively. The experiments were divided into experiment 1 (Ct, M1, and M3) and experiment 2 (Ex1, Ex3, ME1, and ME3).

After the housing familiarization period, when they were from 76 to 89 days old, all of the rats were adapted to aquatic environments and swimming according to a protocol from Lima et al.^36^ that focused on the exposure time in water (5–20 min), water depth (10–80 cm), and load weight (0 or 3% of the body mass). The swimming protocol was individualized in cylindrical and opaque tanks that were 100 cm in height (80 cm in water depth) and 30 cm in diameter; the water temperature was maintained at 31 ± 1 °C in accordance with the guidelines of the American Physiological Society^37^. When they were 90 days old, all animals were submitted to an incremental test (IT) to determine the intensity of the effort corresponding to the individuals’ maximal aerobic capacities.

**Figure 1:**
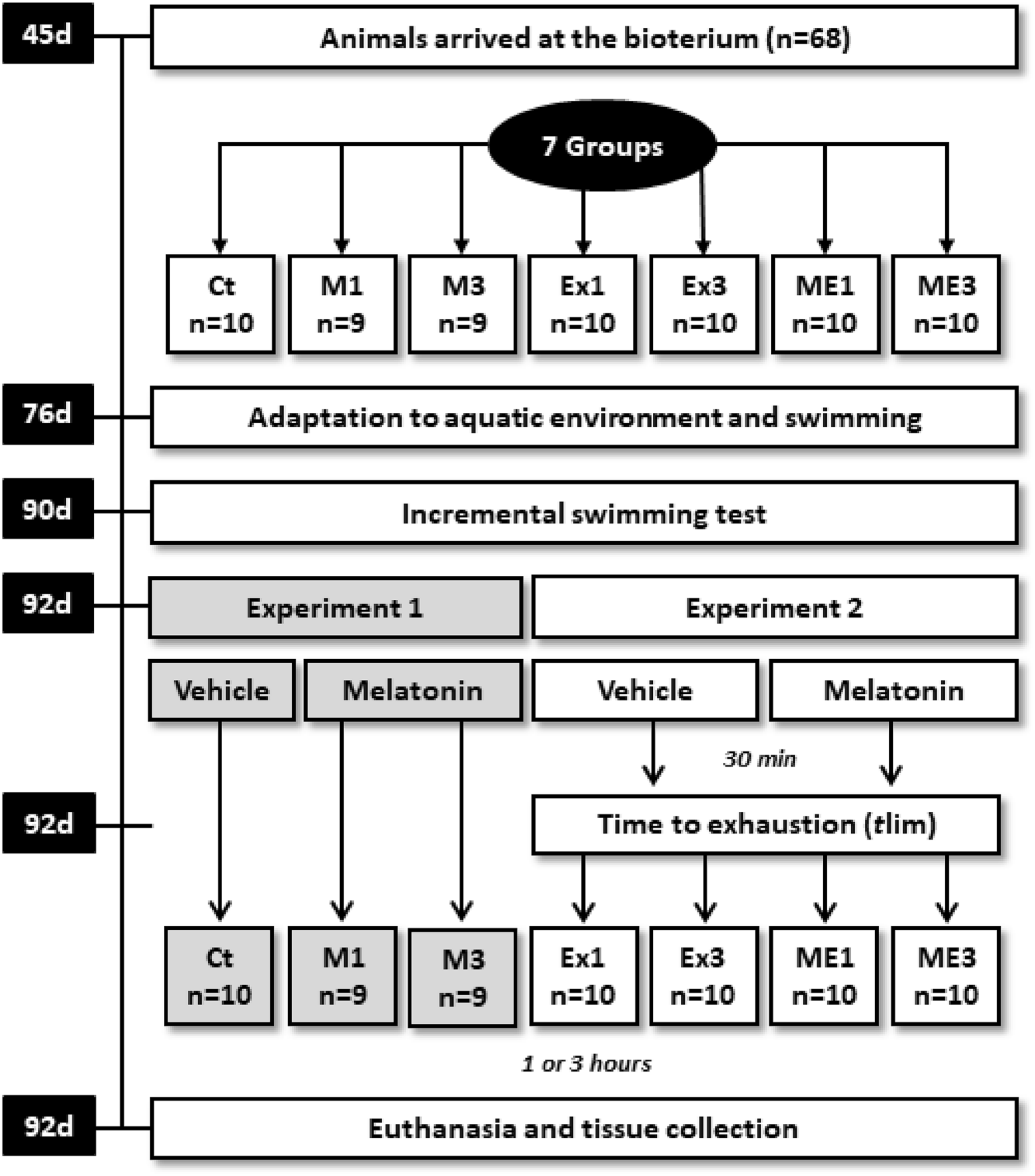
Chronological sequence of events that occurred during the experiments. Control group (Ct); rats treated with melatonin and euthanized 1 h (M1) or 3 h after the last procedures (M3); rats that exercised and were euthanized 1 h (Ex1) or 3 h after *t*lim (Ex3); rats that were treated with melatonin, exercised, and were euthanized 1 h (ME1) or 3 h after *t*lim (ME3). d: days; min: minutes.

At 92 days old, the animals (body mass: 398.06 ± 3.80 g at the end of the experiment) received melatonin or vehicles from 30 min before the swimming exercise until exhaustion at the intensity corresponding to the maximal aerobic capacity, which was called the time to exhaustion (*t*lim). The criteria for identifying the animals’ exhaustion were standardized according to Beck and Gobatto^35^; an analysis of the swimming behaviors of the animals was performed to observe the execution of vigorous efforts in returning to the surface without success for a period of 15 s. The achievement of exhaustion was accepted upon the agreement of two experienced observers using the above criteria. Then, the animals were euthanized 1 or 3 h after the end of the experiment via decapitation, a method that is allowed by the American Veterinary Medical Association^38^. The experimental design is shown in Fig. 1.

### Incremental swimming test

The IT consisted of proportional increases in the load over time in order to identify a disproportionate increase in the concentration of blood lactate at a given moment^39^, which was called the maximal aerobic capacity. Therefore, the animals were subjected to five-minute stages with overloads corresponding to 4.0, 4.5, 5, 5.5, 6, 6.5, and 7.0% of their body mass (% BM); these overloads were attached to the animals’ chests with an elastic strap. After each stage, blood samples (25 µL) were collected from the distal part of the animals' tails and then stored (4 °C) in order to determine the lactate concentration. After analyzing the lactate concentration with the enzymatic method, the intensity of the exercise in relation to the blood lactate concentration was plotted on a scatter plot, and any changes in the blood lactate concentration were identified through visual inspection, as previously described by Matsumoto et al.^40^. Then, two linear regressions were constructed after the breaking point. The intersection of these linear regressions was interpolated to the X-axis and then used to define the intensity corresponding to the anaerobic lactacidemic threshold^39^. The interpolation for line y corresponded to the blood lactate concentration at the intensity of the maximal aerobic capacity.

### Melatonin administration

Melatonin (Sigma Aldrich Chemical Corporation; St Louis, MO, USA; M-5250, > 98%) was dissolved in ethanol (< 0.1%) and diluted in saline (0.9% NaCl) for administration at 10 mg·kg^−1^^14,15^. The preparation was carried out just prior to its use, and it was stored in an amber bottle that was wrapped in aluminum foil. Administration was intraperitoneal and took place 30 min prior to the *t*lim.

### Analytical procedures on biological materials

#### Plasma and serum parameters

During the IT, blood samples (25 μL) were collected from the animals' tails in heparinized and calibrated glass capillaries. These samples were immediately transferred to 1.5 mL tubes containing 400 μL of trichloroacetic acid (4%), which were then agitated and stored at 4 °C. After stirring and centrifuging (3000 rpm for 3 min), 50 µL of supernatant was extracted and transferred to a 96-well microplate, where added 250 µL of reactive solution that was prepared for immediate use (glycine/EDTA and hydrazine hydrate stock), NAD (β-nicotinamide adenine dinucleotide), and LDH (L-lactic dehydrogenase bovine heart) were added; the pH was properly adjusted to 9.45 before the added of NAD and LDH. The samples and reagent were incubated (20 min, 37 °C) and the absorbance was determined in a spectrophotometer (Spectramax i3, Molecular Devices; San José, CA, USA) at 340 nm. The blood lactate concentration was determined in relation to the standard curve constructed from the serial dilution of L-Lactate 1–15 mmol/L.

After euthanasia, an aliquot of approximately 2.0 mL of blood was obtained and allowed to rest for 20 min (4 °C) before a subsequent centrifugation (15 min, 3000 rpm, 10 °C). These samples were stored at − 20 °C for further analysis.

For the glucose analysis, 3 µL of serum was mixed with the kit reagent (300 µL; LaborLab; Guarulhos, SP, Brazil) and incubated for 25 min (25 °C); GOD (≥ 15 kU/L), POD (≥ 2 kU/L), 4-AAT (0.5 mmol/L), phosphates (pH = 7.5, 250 mmol/L), and phenol (5 mmol/L). The glucose absorbance was determined in a spectrophotometer (SpectraMax i3, Molecular Devices; San José, CA, USA) at 505 nm according to the kit’s guidelines.

To determine the triglyceride concentration, 3 µL of serum was mixed with the kit reagent (300 µL; LaborLab; Guarulhos, SP, Brazil) and incubated for 20 min (25 °C); good (pH = 6.8, 50 mmol/L), chlorophenol (2 mmol/L), lipoprotein lipase (≥ 800 U/L), GK (≥ 500 U/L), GPO (≥ 1500 U/L), POD (≥ 900 U/L), ATP (2 mmol/L), and 4-AF (0.4 mmol/L). The triglyceride absorbance was determined in a spectrophotometer (SpectraMax i3, Molecular Devices; San José, CA, USA) at 505 nm according to the kit’s guidelines.

#### Skeletal muscle glycogen

The procedure was performed according to the method presented by Dubois et al.^41^. Firstly, skeletal muscle tissue (200–250 mg; gluteus maximus) was digested in potassium hydroxide (KOH 30%). Then, a saturated sodium sulfate solution (20 µL, Na_2_SO_4_) and ethanol (3 mL, CH_3_CH_2_OH 70%) were added for the precipitation of glycogen. The samples were submitted to the colorimetric phenol (10 µL, C_6_H_6_O) and sulfuric (2.0 mL, H_2_SO_4_) method and measured via spectrophotometry (Hach Company, Loveland, Colo, USA; 490 nm) against a standard glucose curve.

#### Skeletal muscle triglyceride

Initially, skeletal muscle tissue (100–200 mg; gluteus maximus) and Triton X-100 (1%) were mixed at the same proportions (200 mg of tissue to 1 mL of Triton). Next, the samples were homogenized with magnet bars (5 × 3 mm) overnight (2–8 °C) and centrifuged (10 min, 4000 rpm). After this period, 10 µL of the supernatant was extracted, pipetted into a 96-well microplate in a mixture with the kit reagent (200 µL; LaborLab; Guarulhos, SP, Brazil), and incubated for 20 min (25 °C); good (pH = 6.8, 50 mmol/L), chlorophenol (2 mmol/L), lipoprotein lipase (≥ 800 U/L), GK (≥ 500 U/L), GPO (≥ 1500 U/L), POD (≥ 900 U/L), ATP (2 mmol/L), and 4-AF (0.4 mmol/L). The triglyceride absorbance was determined in a spectrophotometer (SpectraMax i3, Molecular Devices; San José, CA, USA) at 505 nm according to the kit’s guidelines.

#### Histological and immunofluorescence procedures

Immediately after euthanasia, the soleus muscle was dusted in talc, frozen in liquid nitrogen, and stored (− 80 °C). Transversal histological frozen Sections (6 μm) were obtained from a cryostat (− 25 °C; Leica CM 1850 UV) and collected on glass slides (26 × 76 mm). Prior to the immunofluorescence protocol, the slides were stained with Hematoxylin–Eosin (HE) in order to identify morphological changes in the tissue that could compromise the analysis with a light microscope.

For the quantification of GLUT4 and FAT/CD36, the sections were double-stained with laminin (for the purpose of demarcating the cells). The slides were incubated with a mix of primary anti-mouse monoclonal antibodies for GLUT4 (dilution 1:1600; Santa Cruz Biotechnology, INC; Dallas, Texas, USA) or for FAT/CD36 (dilution 1:400; Santa Cruz Biotechnology, INC; Dallas, Texas, USA), in combination with anti-rabbit laminin (dilution 1:200; Abcam; Ab11575; Cambridge, UK) diluted in 1% BSA (Bovine Serum Albumin – Sigma Aldrich Chemical Corporation, St Louis, MO, USA), for 45 min at 37 °C. After this period, the sections were washed in PBS solution (three cycles of 5 min), and a mix of secondary antibodies was added: Alexa 488 IgG^1^ to mark GLUT4 with a green color (dilution 1:1000; Jackson ImmunoResearch, Laboratories, INC.; West Grove, PA, USA) or Alexa 594 IgM to mark FAT/CD36 with a red color (dilution 1:1000; Jackson ImmunoResearch, Laboratories, INC.; West Grove, PA, USA), in combination with Alexa Fluor 647 IgG (dilution 1:200; Invitrogen; Carlsbad, California, USA) to mark laminin with a red color or Alexa Fluor 488 IgG to mark laminin with a green color (dilution 1:200; Invitrogen; Carlsbad, California, USA); this procedure was performed for 35 min at 37 °C. The sections were washed again with PBS solution (3 cycles of 5 min) and mounted with FluoroQuest™ Mounting Medium (AAT Bioquest®, INC, Sunnyvale, CA, USA).

The slides were photographed with an automated high-resolution epifluorescent microscopy system (ImageXpress® Micro, Molecular Devices; San José, CA, USA) using an objective lens with a magnification of 20×, with specific filters for GLUT4 (FITC: 1000–1200 ms exposure), FAT/CD36 (Cy5: 1800–2200 ms exposure), and laminin (FITC and Cy5: 200 ms exposure). The images were saved with an identical size and resolution.

The integrated density of the fluorescence intensity of GLUT4 and FAT/CD36 was quantified in five distinct and random fields (height: 220 and width: 220) by the ImageJ 1.52a software (National Institutes of Health, USA), and the images were individually analyzed. The mean values of the proteins in each sample were calculated and plotted in a graph.

### Statistical data analysis and processing

The data were presented as mean ± standard error. Normality was verified with the Shapiro–Wilk test (p > 0.05). The time to exhaustion was analyzed with the *t*-test for independent samples by using pooled data from all exercised animals that were treated with melatonin (ME1 and ME3) versus exercised animals that were treated with a vehicle (Ex1 and Ex3). A one-way analysis of variance was performed for all the parameters of experiment 1 (experiment 1: Ct, M1, and M3) and for lactacidemia and % BM in experiment 2. A two-way analysis of variance was performed for the other parameters in experiment 2—the effects of melatonin (melatonin or vehicle) and the time of euthanasia (1 or 3 h) (experiment 2: Ex1, Ex3, ME1, and ME3). When appropriate, we used the Newman–Keuls post hoc test. A significance level of 5% was established for all analyses, and Statistica 7.0 (StatSoft, Inc.; Tulsa, OK, USA) was used. The effect size (ES) analysis was used as a complementary test. The thresholds for small, moderate, and large effects were 0.20, 0.50, and 0.80, respectively. The ES was determined with the formula: (mean1–mean2)/pooled SD^42^.

## Results

### Experiment 1—acute adminstration of melatonin increases glycogen content and GLUT4 in resting skeletal muscle

#### Energy substrates in the muscle and blood

There was an increase in glycogen content in the M1 and M3 groups with respect to the Ct group (F = 8.85, p < 0.01). No differences among the groups were found for the muscle triglycerides, blood glucose, or blood triglyceride (F = 1.06, F = 3.11, and F = 0.36, respectively; p > 0.05) (Table 1). Large effects were demonstrated when comparing Ct with M1 (p = 0.021, ES: 1.59) and Ct with M3 (p = 0.001, ES: 2.12) in terms of the muscle glycogen content.

**Table 1:** Data on the muscle glycogen and triglyceride content and blood glucose and triglyceride concentrations in the groups that were treated with the vehicle (Ct) or melatonin (M1 and M3) and euthanized 1 (M1) or 3 (M3) hours after the last procedures.

	Ct	M1	M3
Muscle glycogen (mg·g^-1^)	4.97 ± 0.01	6.08 ± 0.02^a^	6.84 ± 0.03^a^
Muscle triglyceride (mg·g^-1^)	1.16 ± 0.04	1.12 ± 0.02	1.19 ± 0.01
Blood glucose (mg·dL^-1^)	132.69 ± 6.43	153.17 ± 6.67	145.01 ± 4.10
Blood triglyceride (mg·dL^-1^)	96.04 ± 5.10	91.28 ± 4.60	98.62 ± 7.90

Control group (Ct); rats treated with melatonin and euthanized 1 h (M1) or 3 h after the last procedures (M3). Values are expressed as mean and standard error. ^a^p < 0.05 with respect to Ct for the same parameter. g: grams; mg: milligrams; dL: deciliters.

#### Muscle GLUT4 and FAT/CD36

**Figure 2:**
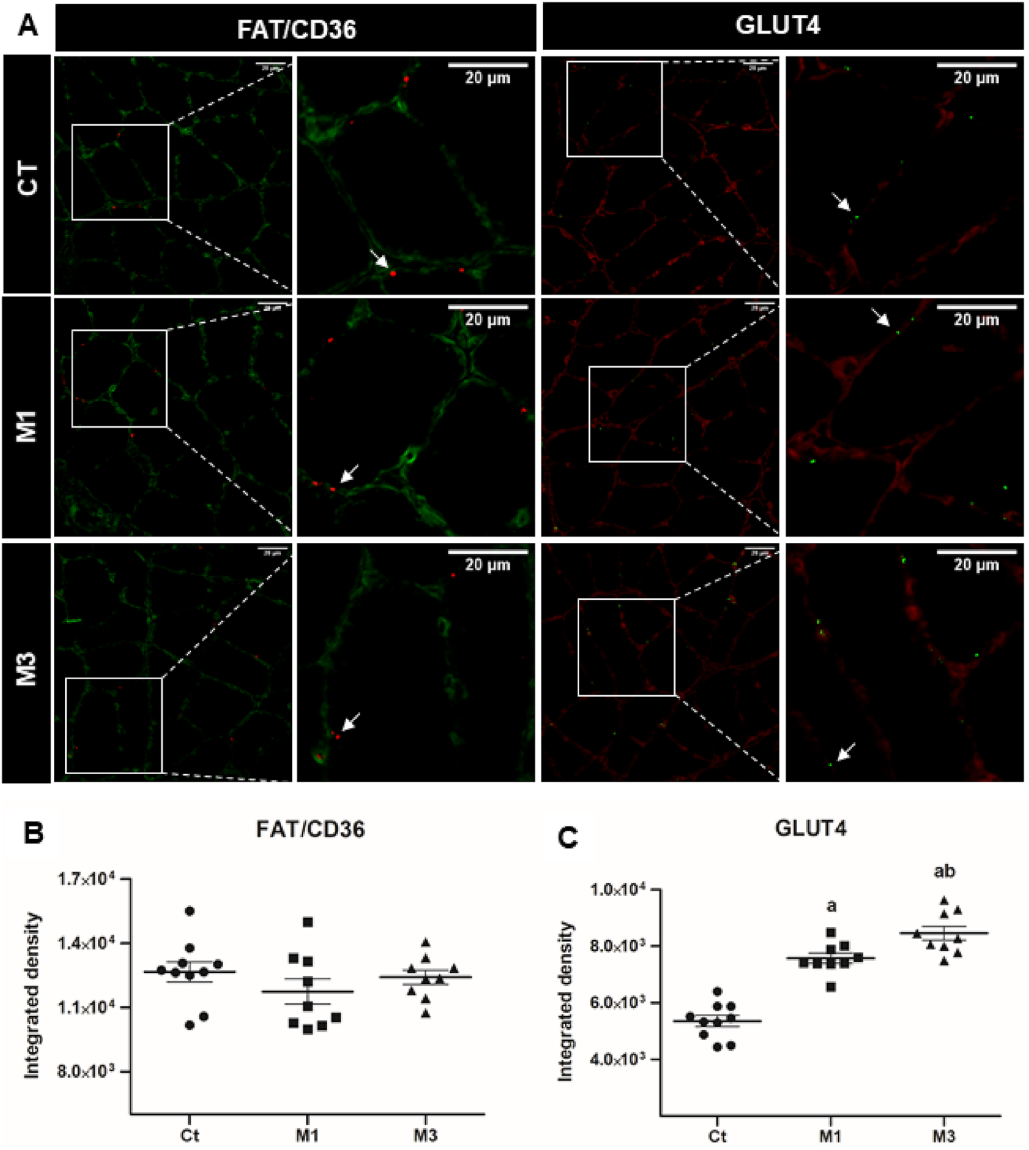
GLUT4 and FAT/CD36 in skeletal muscles. Representative samples of laminin (green) with FAT/CD36 (red) in the soleus skeletal muscle with immunofluorescence (**A**). Representative samples of laminin (red) with GLUT4 (green) in the soleus skeletal muscle with immunofluorescence (**A**) in the control group (Ct) and rats treated with melatonin and euthanized 1 h (M1) or 3 h after the last procedures group (M3). The white arrows indicate FAT/CD36 and GLUT4 in the soleus skeletal muscle. The figures represent the means and standard errors of the FAT/CD36 (**B**) and of the GLUT4 (**C**). ^a^p < 0.05 with respect to Ct; ^b^p < 0.05 with respect to M1 for the same parameter. For the illustration, an objective lens = 20 × was used; bars = 20 µm; zoom = 300 height and 300 width.

GLUT4 increased in the M1 and M3 groups compared to the Ct group (F = 60.70, p < 0.05). Otherwise, no differences among the groups were observed for FAT/CD36 (F = 0.99, p > 0.05) (Fig. 2). Large effects were demonstrated when comparing Ct with M1 (p = 0.0001, ES: 3.84), Ct with M3 (p = 0.0001, ES: 4.57), and M1 with M3 (p = 0.0006, ES: 1.39) in terms of muscle GLUT4.

### Experiment 2—melatonin improves performance and accelerates metabolic recovery in skeletal muscle

#### Incremental test and time to exhaustion at maximal aerobic capacity

As expected, in the ITs, lactacidemia and the percentage of body mass (% BM) did not show differences when comparing all seven groups (Ct: 4.14 ± 0.24; M1: 3.39 ± 0.28; M3: 3.56 ± 0.40; Ex1: 3.71 ± 0.22; Ex3: 3.88 ± 0.37; ME1: 3.96 ± 0.27; ME3: 4.06 ± 0.20 mM and Ct: 5.42 ± 0.26; M1: 5.41 ± 0.15; M3: 5.56 ± 0.20; Ex1: 5.11 ± 0.18; Ex3: 5.30 ± 0.15; ME1: 5.81 ± 0.14; ME3: 5.46 ± 0.15% BM; F = 0.69 and F = 1.49, respectively; p > 0.05). Regarding *t*lim, melatonin increased the performance (ME1 and ME3; 78.30 ± 9.36 min; p = 0.01, ES: 0.99, 49.42%) compared to the performance with the vehicle (Ex1 and Ex3; 52.40 ± 5.25 min).

#### Energy substrates in the muscles and blood

The glycogen content increased in the gluteus maximus at 3 h compared to that at 1 h (F = 15.57, p < 0.01; 3 h > 1 h), while the groups that exercised and received melatonin did not experience a difference in glycogen content compared to the animals that received the vehicle (F = 1.12, p = 0.72). For the muscle triglyceride, the time and treatment did not promote an effect on the gluteus maximus (F = 0.70, p = 0.40 and F = 0.05, p = 0.80, respectively) (Table 2). Large effects on the glycogen content were demonstrated when comparing Ex1 with ME3 (p = 0.041, ES: 2.09), Ex3 with ME1 (p = 0.012, ES: 1.25), and ME1 with ME3 (p = 0.004, ES: 3.09). Large effects on the triglyceride content were observed when comparing Ex1 with Ex3 (p = 0.019, ES: 1.74), Ex1 with ME1 (p = 0.007, ES: 1.34), Ex3 with ME3 (p = 0.018, ES: 1.84), and ME1 with ME3 (p = 0.004, ES: 1.42).

**Table 2:** Data on the muscular glycogen and triglyceride content, as well as the blood glucose and triglyceride concentrations, in the exercised groups (Ex1, Ex3, ME1, and ME3), those treated with the vehicle (Ex1 and Ex3) or melatonin (ME1 and ME3), and those euthanized 1 (Ex1 and ME1) or 3 (Ex3 and ME3) hours after *t*lim.

	Ex1	Ex3	ME1	ME3
Muscle glycogen (mg·g^-1^)	4.19 ± 0.03	5.44 ± 0.06	3.49 ± 0.03^b^	5.82 ± 0.01^ac^
Muscle triglyceride (mg·g^-1^)	1.16 ± 0.04	1.36 ± 0.03^a^	1.43 ± 0.08^a^	1.12 ± 0.04^bc^
Blood glucose (mg·dL^-1^)	108.27 ± 4.22	138.21 ± 8.71^a^	122.81 ± 8.01	106.96 ± 7.94^b^
Blood triglyceride (mg·dL^-1^)	126.14 ± 4.05	106.82 ± 3.56^a^	123.97 ± 3.52^b^	84.35 ± 4.72^abc^

Rats that exercised and were euthanized 1 h (Ex1) or 3 h after *t*lim (Ex3); rats that were treated with melatonin, exercised, and were euthanized 1 h (ME1) or 3 h after *t*lim (ME3). Values are expressed as the mean and standard error. ^a^p < 0.05 with respect to Ex1; ^b^p < 0.05 with respect to Ex3; ^c^p < 0.05 with respect to ME1 for the same parameter. g: grams; mg: milligrams; dL: deciliters.

The blood triglyceride concentration was higher at 1 h than that at 3 h (F = 54.39, p < 0.01; 3 h < 1 h), while the blood glucose remained unchanged between the animals that were euthanized 1 or 3 h after *t*lim (F = 0.82, p = 0.36). Moreover, melatonin decreased the serum triglyceride concentration (F = 9.50, p < 0.01), but did not cause a change in the blood glucose compared to animals that received the vehicle (F = 1.16, p = 0.28) (Table 2). Large effects on the blood glucose concentration were demonstrated when comparing Ex1 with Ex3 (p = 0.026, ES: 1.52) and Ex3 with ME3 (p = 0.035, ES: 1.19). Large effects on the blood triglyceride concentration were noted when comparing Ex1 with Ex3 (p = 0.004, ES: 1.69), Ex3 with ME1 (p = 0.004, ES: 1.61), Ex1 with ME3 (p = 0.0001, ES: 3.17), Ex3 with ME3 (p = 0.0004, ES: 1.81), and ME1 with ME3 (p = 0.0001, ES: 3.20).

#### Muscle GLUT4 and FAT/CD36

**Figure 3:**
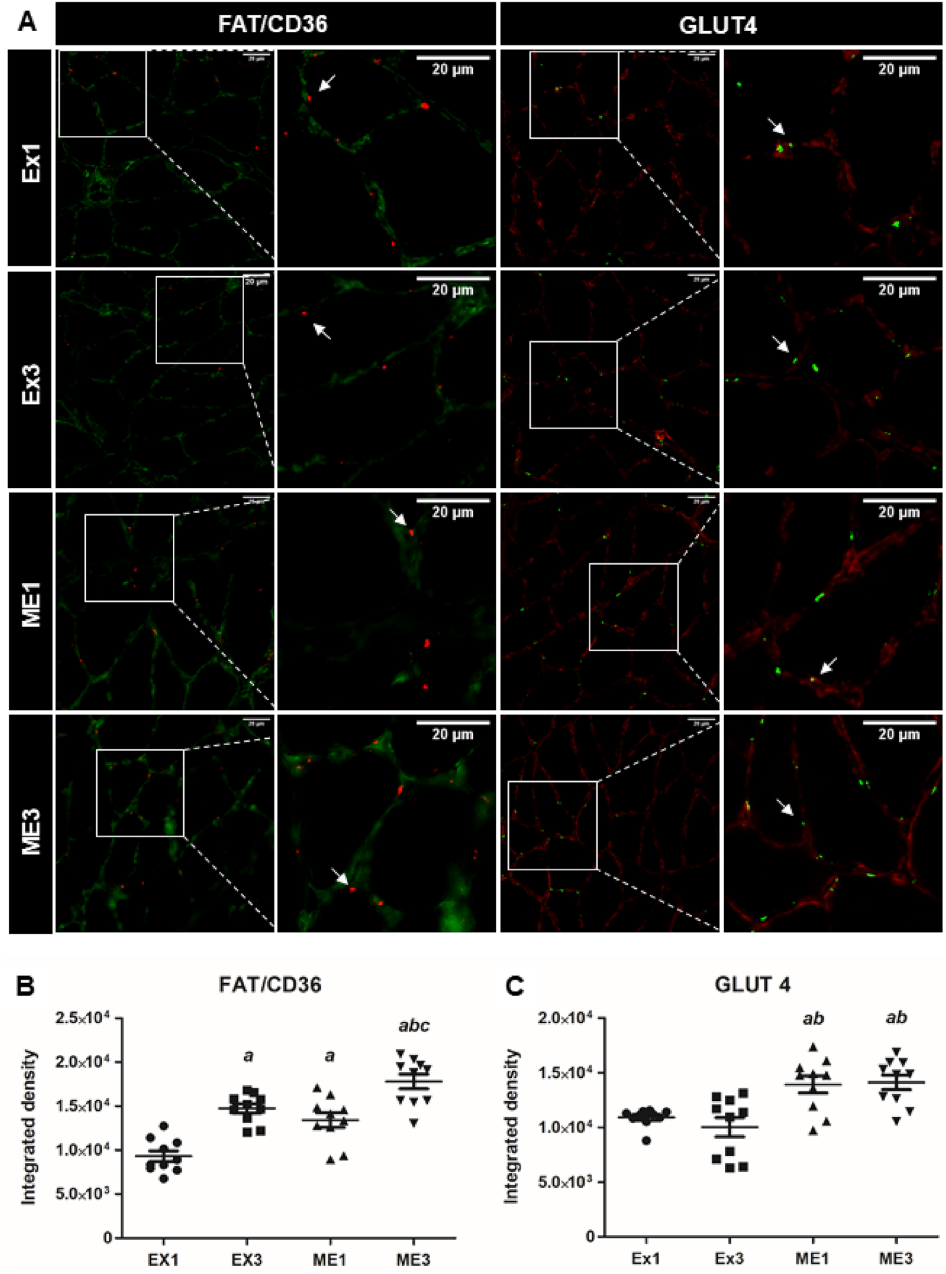
GLUT4 and FAT/CD36 in the skeletal muscle. Representative samples of laminin (green) with FAT/CD36 (red) in the soleus skeletal muscle with immunofluorescence (**A**). Representative samples of laminin (red) with GLUT4 (green) in the soleus skeletal muscle with immunofluorescence (**A**) in rats that exercised and were euthanized 1 h (Ex1) or 3 h after *t*lim (Ex3) and rats that were treated with melatonin, exercised, and were euthanized 1 h (ME1) or 3 h after *t*lim (ME3). The white arrows indicate FAT/CD36 and GLUT4 in the soleus skeletal muscle. The figures show the means and standard errors of FAT/CD36 (**B**) and of GLUT4 (C). ^a^p < 0.05 with respect to Ex1; ^b^p < 0.05 with respect to Ex3; ^c^p < 0.05 with respect to ME1 for the same parameter. For the illustration, objective lens = 20 × was used; bars = 20 µm; zoom = 300 height and 300 width.

Melatonin increased GLUT4 (F = 26.83, p < 0.01) without a time effect (F = 0.25, p = 0.61). Furthermore, the effects of melatonin and time (3 h > 1 h) increased FAT/CD36 (F = 25.28, p < 0.01 and F = 47.56, p < 0.01, respectively) (Fig. 3). Large effects on GLUT4 were demonstrated when comparing Ex1 with ME1 (p = 0.003, ES: 1.85), Ex3 with ME1 (p = 0.001, ES: 1.49), Ex1 with ME3 (p = 0.006, ES: 2.20), and Ex3 with ME3 (p = 0.001, ES: 1.68). Large effects on FAT/CD36 were obtained when comparing Ex1 with Ex3 (p = 0.0001, ES: 3.05), Ex1 with ME1 (p = 0.0003, ES: 1.80), Ex1 with ME3 (p = 0.0001, ES: 3.76), Ex3 with ME3 (p = 0.004, ES: 1.43), and ME1 and ME3 (p = 0.0004, ES: 1.67).

## Discussion

The main finding of this study was the ability of melatonin to increase GLUT4, FAT/CD36, and the metabolic recovery process in exercised skeletal muscle favoring cellular environment for future efforts, which corroborated our hypothesis. In addition, this is the first study to highlight the acute effect of melatonin administration on energy substrate transporters, as well as melatonin’s role in the metabolic recovery of rats that were submitted to an individualized exhaustive exercise session with an intensity corresponding to maximal aerobic capacity.

In experiment 1, we observed that the acute administration of melatonin increased the muscular glycogen content (p < 0.05; 22.33% and 37.62%, M1 and M3 > Ct, respectively). This was associated with an increase in the GLUT4 presented by the animals that were treated with melatonin compared to the animals treated with the vehicle (p < 0.05; 41.87% and 57.87%, M1 and M3 > Ct, respectively). It is well known that melatonin acts by binding to membrane receptors that are coupled to G proteins (MTNR1A or MT1 and MTNR1B or MT2)^43^, which are present in the membranes of skeletal muscles (BioGPS (http://biogps.gnf.org)); this causes an increase in the activity of IRS-1 and PI3K^44^. These upstream signals are responsible for raising the activity and content of GLUT4 in a way that is similar to insulin signaling.

Considering the robust effect of melatonin on muscular glycogen content (as observed in experiment 1) and the importance of the oxidation of carbohydrates during exercise, we investigated the effects of melatonin in animals that exercised at their maximal aerobic capacity and were euthanized at different times after the exercise. Therefore, the intensity of the effort was individually determined by using an incremental test; no differences were demonstrated between the groups (p > 0.05) for lactacidemia or % BM before the *t*lim. Then, the ergogenic capacity of melatonin was confirmed by the time to exhaustion (*t*lim) (Ex1 and Ex3, 52.40 ± 19.66 min; ME1 and ME3, 78.30 ± 32.43 min; p = 0.01). These findings corroborate those of previous studies published by our group, which demonstrated high performance in *t*lim by animals treated with melatonin during periods of wakefulness (10 mg·kg^−1^)^14,15^.

According to Bergstrom et al.^45^, an increased glycogen content is one of the main determinants for performance in moderate and prolonged exercise. In addition, the dependence on carbohydrates in high-intensity and long-term physical exercises is well recognized^46^. To confirm this, previous studies by our group demonstrated that, when submitted to endurance exercise until exhaustion at individualized intensities of effort corresponding to the maximal aerobic capacity, Wistar rats (92 days old) showed a depletion of the glycogen content in the gluteus maximus immediately after swimming exercise, among other effects (p < 0.05)^47^. In addition, Matsui et al.^48^ demonstrated that as the duration of the exercise increased, the glycogen content was observed to decrease after the effort. Based on these assumptions, animals treated with melatonin were expected to show lower values for the muscular glycogen content, as they swam longer than animals treated with the vehicle (p = 0.01, 49.42%). However, in the analysis of the muscular glycogen content, despite the ME1 group showing a reduction with respect to the Ex1 group (16.7%), the values were found to be statistically equal (p > 0.05). This possibly occurred due to the ability of melatonin to increase the glycogen content (p < 0.05; M1 and M3 > Ct), thus improving the rats’ performance in the exercise. Therefore, the data indicated that melatonin is one of the factors responsible for the better performance due to the increase in the pre-effort glycogen stores (as seen in the M1 and M3 groups), thus consequently increasing the time until exhaustion during the swimming exercise at an intensity corresponding to the maximal aerobic capacity (as seen in the ME1 and ME3 groups).

Regarding the metabolic recovery, in the presence of melatonin, the animals euthanized 3 h after *t*lim (ME3) showed an increase in glycogen content with respect to the animals euthanized 1 h after *t*lim (ME1) (p < 0.05; 40.03%). However, in the absence of melatonin, no differences in glycogen content were demonstrated when comparing the Ex3 and Ex1 groups (p > 0.05). Moreover, no statistical differences were observed between the ME3 and Ex3 groups (p > 0.05); however, the ME3 group swam longer than the Ex3 group (p < 0.05). Based on these results, in the presence of melatonin, the metabolic recovery after exercise until exhaustion was improved. The enhancement of the glycogen content possibly occurred due to the increase in the GLUT4 demonstrated by the animals treated with melatonin (ME1 and ME3) in comparison to the animals treated with the vehicle (Ex1 and Ex3, p < 0.05), thus increasing the uptake of glucose in the skeletal muscles after exercise. These data are consistent with the findings of Mendes et al.^49^, who demonstrated an increase in the content of PI3K, GLUT4, and glycogen stores in the skeletal muscles of rats that were submitted to treadmill training (20 m min^-1^, 5 days week^−1^, 16 weeks) and treated with melatonin (10 mg·kg day^−1^, 8 weeks).

Regarding the metabolism of lipids, the muscular triglyceride content of the ME1 group was higher than that of the Ex1 group (p < 0.05), which was possibly due to the increase in the FAT/CD36 shown by the ME1 group compared to Ex1 group (p < 0.05). Interestingly, in the absence of melatonin, such an increase with respect to the Ex1 group (p < 0.05) occurred only 3 h after the *t*lim (Ex3). Due to the increase in FAT/CD36 in comparison to Ex1 (p < 0.05), it also occurs only 3 h after the *t*lim (Ex3). Therefore, melatonin enhances the triglyceride content 1 h after exercise, which possibly improves the metabolic recovery process. Assuming that the activation pathway of FAT/CD36 is similar to that of GLUT4^50,51^ and given the presence of MTNR1A/MTNR1B in the skeletal muscles of rats^44^ (BioGPS (http://biogps.gnf.org)), we believe that the enhancement of FAT/CD36 was possibly influenced by melatonin through its binding to the MTNR1B receptor and, consequently, its activation of PI3K, IRS^44^, DAG, IP3, PLC, and^43^ Ca^2+^. However, there are no studies concerning the effects of acute melatonin administration on the content of FAT/CD36 in exercised skeletal muscles. Furthermore, the ME3 group showed a reduction in the muscle triglyceride content in comparison to the ME1 group (p < 0.05), which was possibly due to the greater use of fat while resting, which is considered an optimal muscle environment for fat oxidation and the consequent supply of ATPs for the post-exercise recovery. In addition, the reduction demonstrated by the ME3 group possibly occurred due to the increase in FAT/CD36 in the ME3 group (p < 0.05), which consequently increased the transport of triglyceride from the blood to the skeletal muscles to be oxidized. Thus, it would be plausible to affirm the ability of melatonin to accelerate the metabolic recovery processes related to carbohydrate metabolism and to modulate the supply of lipids after exhaustive exercise.

Some limitations in this manuscript must be addressed. First, other dosages should be tested in order to demonstrate the lowest concentration of melatonin that would make it possible to achieve similar effects. Finally, we focused on transporters and their respective substrates; however, evaluating the activation of upstream signals would be quite enlightening. However, our findings make clear that future studies must be conducted in order to deepen the knowledge on this relevant area.

In conclusion, the present study demonstrated that melatonin increased the availability of glycidic substrates and GLUT4 in skeletal muscles and consequently provided a greater tolerance to physical exercise. In addition, melatonin improved the efficiency of the recompositing of energetic substrates and enhanced GLUT4 and FAT/CD36 in the exercised skeletal muscles, thus improving the cellular environment for future efforts, at least from the bioenergetic point of view.

## References

[1] Reiter RJ, Tan DX, Fuentes-Broto L (2010). Melatonin: a multitasking molecule. Prog. Brain Res..

[2] Reiter RJ (2016). Melatonin as an antioxidant: under promises but over delivers. J. Pineal Res..

[3] Manchester LC (2015). Melatonin: an ancient molecule that makes oxygen metabolically tolerable. J. Pineal Res..

[4] Galano A, Tan DX, Reiter RJ (2018). Melatonin: a versatile protector against oxidative DNA damage. Molecules.

[5] Rodriguez C (2004). Regulation of antioxidant enzymes: a significant role for melatonin. J. Pineal Res..

[6] Mauriz JL, Collado PS, Veneroso C, Reiter RJ, González-Gallego J (2013). A review of the molecular aspects of melatonin’s anti-inflammatory actions: recent insights and new perspectives. J. Pineal Res..

[7] Cipolla-Neto J, Amaral FG, Afeche SC, Tan DX, Reiter RJ (2014). Melatonin, energy metabolism, and obesity: a review. J. Pineal Res..

[8] Zare H, Shafabakhsh R, Reiter RJ, Asemi Z (2019). Melatonin is a potential inhibitor of ovarian cancer: molecular aspects. J. Ovarian Res..

[9] Li Y (2017). Melatonin for the prevention and treatment of cancer. Oncotarget.

[10] Gunata MEHMET, Parlakpinar HAKAN, Acet HA (2019). Melatonin: a review of its potential functions and effects on neurological diseases. Revue Neurol..

[11] Sharma S, Singh H, Ahmad N, Mishra P, Tiwari A (2015). The role of melatonin in diabetes: therapeutic implications. Arch. Endocrinol. Metab..

[12] Xie Z (2017). A review of sleep disorders and melatonin. Neurol. Res..

[13] Xu P (2017). Melatonin prevents obesity through modulation of gut microbiota in mice. J. Pineal Res..

[14] Beck WR, Botezelli JD, Pauli JR, Ropelle ER, Gobatto CA (2015). Melatonin has an ergogenic effect but does not prevent inflammation and damage in exhaustive exercise. Sci. Rep..

[15] Beck WR, Scariot PPM, Gobatto CA (2016). Melatonin is an ergogenic aid for exhaustive aerobic exercise only during the wakefulness period. Int. J. Sports Med..

[16] Hargreaves M, Spriet LL (2020). Skeletal muscle energy metabolism during exercise. Nat. Metab..

[17] Klip A, McGraw TE, James DE (2019). Thirty sweet years of GLUT4. J. Biol. Chem..

[18] Richter EA (2021). Is GLUT4 translocation the answer to exercise-stimulated muscle glucose uptake?. Am. J. Physiol. Endocrinol. Metab..

[19] Chibalin AV (2000). Exercise-induced changes in expression and activity of proteins involved in insulin signal transduction in skeletal muscle: differential effects on insulin-receptor substrates 1 and 2. Proc. Natl. Acad. Sci..

[20] Ren JM, Semenkovich CF, Gulve EA, Gao J, Holloszy JO (1994). Exercise induces rapid increases in GLUT4 expression, glucose transport capacity, and insulin-stimulated glycogen storage in muscle. J. Biol. Chem..

[21] Goodyear LJ, Hirshman MF, Smith RJ, Horton ES (1991). Glucose transporter number, activity, and isoform content in plasma membranes of red and white skeletal muscle. Am. J. Physiol. Endocrinol. Metab..

[22] Burke LM, Hawley JA (2018). Swifter, higher, stronger: What’s on the menu?. Sci..

[23] Watt MJ, Heigenhauser GJ, Dyck DJ, Spriet LL (2002). Intramuscular triacylglycerol, glycogen and acetyl group metabolism during 4 h of moderate exercise in man. J. Physiol..

[24] Holloszy JO, Kohrt WM, Hansen PA (1998). The regulation of carbohydrate and fat metabolism during and after exercise. Front. Biosci..

[25] Smith BK, Bonen A, Holloway GP (2012). A dual mechanism of action for skeletal muscle FAT/CD36 during exercise. Exerc. Sport Sci. Rev..

[26] Holloway GP (2009). Mitochondrial function and dysfunction in exercise and insulin resistance. Appl. Physiol. Nutr. Metab..

[27] Bonen A (2009). PGC-1α-induced improvements in skeletal muscle metabolism and insulin sensitivity. Appl. Physiol. Nutr. Metab..

[28] Greene NP (2012). Regulators of blood lipids and lipoproteins? PPARδ and AMPK, induced by exercise, are correlated with lipids and lipoproteins in overweight/obese men and women. Am. J. Physiol. Endocrinol. Metab..

[29] Bicer M (2011). Interactive effects of melatonin, exercise and diabetes on liver glycogen levels. Endokrynol. Pol..

[30] Kaya O, Kilic M, Celik I, Baltaci AK, Mogulkoc R (2010). Effect of melatonin supplementation on plasma glucose and liver glycogen levels in rats subjected to acute swimming exercise. Pak. J. Pharm. Sci..

[31] Sánchez-Campos S (2001). Effects of melatonin on fuel utilization in exercised rats: role of nitric oxide and growth hormone. J. Pineal Res..

[32] Mazepa RC, Cuevas MJ, Collado PS, Gonzalez-Gallego J (2000). Melatonin increases muscle and liver glycogen content in nonexercised and exercised rats. Life Sci..

[33] Leckey JJ, Burke LM, Morton JP, Hawley JA (2016). Altering fatty acid availability does not impair prolonged, continuous running to fatigue: evidence for carbohydrate dependence. J. Appl. Physiol..

[34] Krssak M (2000). Intramuscular glycogen and intramyocellular lipid utilization during prolonged exercise and recovery in man: a 13C and 1H nuclear magnetic resonance spectroscopy study. J. Clin. Endocrinol. Metab..

[35] Beck W, Gobatto C (2013). Effects of maximum intensity aerobic swimming exercise until exhaustion at different times of day on the hematological parameters in rats. Acta Physiol. Hung..

[36] Lima AAD (2017). Two water environment adaptation models enhance motor behavior and improve the success of the lactate minimum test in swimming rats. Motriz..

[37] APS. Resource book for the design of animal exercise protocols. *Am. J. Physiol.* **137** (2006).

[38] American Veterinary Medical Association. *AVMA Guidelines on Euthanasia*. AVMA, Schaumber, Illinois (2013).

[39] Zagatto AM, Papoti M, Gobatto CA (2008). Validity of critical frequency test for measuring table tennis aerobic endurance through specific protocol. J. Sports Sci. Med..

[40] Matsumoto I (1999). Effects of swimming training on aerobic capacity and exercise induced bronchoconstriction in children with bronchial asthma. Thorax.

[41] Dubois M, Gilles KA, Hamilton JK, Rebers PT, Smith F (1956). Colorimetric method for determination of sugars and related substances. Anal. Chem..

[42] Cohen D (1988). Statistical Power Analysis for the Behavioral Sciences.

[43] Amaral FGD, Andrade-Silva J, Kuwabara WM, Cipolla-Neto J (2019). New insights into the function of melatonin and its role in metabolic disturbances. Expert. Ver. Endocrinol. Metab..

[44] Ha E (2006). Melatonin stimulates glucose transport via insulin receptor substrate-1/phosphatidylinositol 3-kinase pathway in C2C12 murine skeletal muscle cells. J. Pineal Res..

[45] Bergström J, Hermansen L, Hultman E, Saltin B (1967). Diet, muscle glycogen and physical performance. Acta Physiol. Scand..

[46] Hawley JA, Leckey JJ (2015). Carbohydrate dependence during prolonged, intense endurance exercise. Sports Med..

[47] Beck WF, De Araujo GG, Menezes Scariot PP, Massellidos Reis IG, Gobatto CA (2014). Time to exhaustion at anaerobic threshold in swimming rats: metabolic investigation. Bratisl. Lek. Listy..

[48] Matsui T (2011). Brain glycogen decreases during prolonged exercise. J. Physiol..

[49] Mendes C (2013). Adaptations of the aging animal to exercise: role of daily supplementation with melatonin. J. Pineal Res..

[50] Holloway GP, Luiken JJFP, Glatz JFC, Spriet LL, Bonen A (2008). Contribution of FAT/CD36 to the regulation of skeletal muscle fatty acid oxidation: an overview. Acta Physiol. Scand..

[51] Koonen DP, Glatz JF, Bonen A, Luiken JJ (2005). Long-chain fatty acid uptake and FAT/CD36 translocation in heart and skeletal muscle. BBA Mol. Cell Biol. Lipids.

